# Effects of Non-Lethal High-Temperature Stress on *Bradysia odoriphaga* (Diptera: Sciaridae) Larval Development and Offspring

**DOI:** 10.3390/insects11030159

**Published:** 2020-03-01

**Authors:** Caihua Shi, Seng Zhang, Jingrong Hu, Youjun Zhang

**Affiliations:** 1 Forewarning and Management of Agricultural and Forestry Pests, Hubei Engineering Technology Center, Yangtze University, Jingzhou 434025, Hubei, China; shicaihua1980@126.com (C.S.); cjdxnxyzs@126.com (S.Z.); 2 Department of Plant Protection, Institute of Vegetables and Flowers, Chinese Academy of Agricultural Sciences, Beijing 100081, China

**Keywords:** *Bradysia odoriphaga*, Chinese chives, high temperature, next generation, growth

## Abstract

Throughout China, the dipteran pest *Bradysia odoriphaga* significantly reduces Chinese chive production; therefore, identifying conditions that influence its growth and development is crucial for developing ecological regulation strategies. In this study, different non-lethal high temperatures and treatment durations were used to stress the third-instar larvae of *B. odoriphaga*, and the effects of this treatment on their growth and offspring were recorded and analyzed. The results showed that the average larval mortality increased with increased temperature and prolonged exposure times. After stress treatment at 40 °C for 2 h, 100% of larvae died within 5 days, which was not significantly different from the 5-day average larval mortality (90.66%) after stress at 37 °C for 4 h, but significantly higher than the 5-day average larval mortality (72.00%) after stress at 40 °C for 1 h. After 5 days, all still-living larvae could pupate, and there was no significant difference in average pupal period after pupation. However, the eclosion rate of subsequent pupae decreased with increased temperature and prolonged exposure times, and were only 43.00% and 42.73% after larvae were stressed at 37 °C for 4 h and 40 °C for 1 h, respectively. After eclosion into adults, there was no significant difference in the lifespan of unmated female adults, while the lifespan of unmated male adults was significantly reduced to 1.67 d and 2 d after larvae were stressed at 37 °C for 4 h and 40 °C for 1 h, respectively. However, there was no significant difference in male and female adult longevity after mating. There was no significant difference in oviposition or egg hatchability. This indicates that non-lethal high temperature at 37 °C for 4 h can hinder development and allow control of *B. odoriphaga*. There is great potential for non-lethal high temperature to be applied in the field to control agricultural pests.

## 1. Introduction

Chinese chive (*Allium tuberosum* Rottler ex Sprengel) is an edible and medicinal plant that is widely grown in China, Vietnam, Thailand, Indonesia, Malaysia, and the Philippines [1,2]. The major Chinese chive pest, *Bradysia odoriphaga* Yang and Zhang (Diptera: Sciaridae) [3], is common in China and has a wide host range that includes seven families and 30 plant species, such as garlic, Welsh onion, cabbage, radish, melon, celery, flowers, mushrooms, and Chinese chive [4,5]. *B. odoriphaga* is distributed at a depth of 0–5 cm in the soil [6], and especially damages Chinese chive rhizomes [7]. Uncontrolled, the pest could cause yield losses of up to 50% and destroy entire plants [8].

Many techniques for *B. odoriphaga* control have been investigated, including entomopathogenic nematodes [9,10,11], colored plates, and other methods that do not involve chemical pesticides [12,13,14,15,16]. Each of these methods, however, has limitations, such as slow efficacy and high cost, and *B. odoriphaga* is still a serious threat to Chinese chive production. Therefore, insecticide applications are still the most popular *B. odoriphaga* control methods [17]. Accidental poisoning frequently occurs from eating treated Chinese chives [18]; therefore, an effective, inexpensive, and environmentally safe method is needed to control *B. odoriphaga* on Chinese chive.

The temperature dependence of insect development has been frequently investigated. The impact of temperature on insects may be reflected in their development, fecundity, lifetime, feeding behaviour, etc. For example, extreme daytime maximum temperatures have been shown to hinder the feeding behaviour of the *Leucoptera coffeella*, extend the lifespan of the adult *Grapholitha molesta*, and affect the fecundity of female *Bactrocera dorsalis* and *Spodoptera exigua* [19,20,21]. Night-time warming in the appropriate temperature range resulted in a linear decrease in the survival of the aphids [22]. *Bactrocera cucurbitae* adults tolerate 41–47 °C but temperatures above 51 °C are lethal [23]. In a word, at temperatures slightly above those that promote the fastest rate of development, the fitness of insects rapidly falls with increasing temperature [24]. Thus, artificially raising the temperature of insects in the field is a promising strategy for environmentally-friendly pest control. Recently, Shi et al. [25] reported that 3.7 h of soil solarization at 40 °C will produce 100% control against all *B. odoriphaga* stages. In addition, according to our laboratory studies, the optimal temperature for *B. odoriphaga* growth ranges from 20 to 25 °C [8]. Higher temperatures reduce *B. odoriphaga* survival [26], and *B. odoriphaga* abundance in China is very low in summer [6]. These findings suggest that extremely high temperatures can kill *B. odoriphaga*, and non-lethal high temperatures can hinder their growth or development. Assuming that we raise the soil temperature to some suitable non-lethal high temperature to hinder the growth or development of *B. odoriphaga*, or to control them below the level of economic harm threshold, it will not only reduce the damage to Chinese chives but also protect the ecological balance of the species. Therefore, it is important to research the effect of non-lethal high temperatures on *B. odoriphaga* development for use as a potentially important ecological strategy for *B. odoriphaga* control on Chinese chives. In the current study, we systematically varied non-lethal high temperatures and duration times to stress *B. odoriphaga* larvae and recorded subsequent effects on their development and offspring.

## 2. Materials and Methods

### 2.1. Bradysia Odoriphaga

The *B. odoriphaga* population used in this study was originally obtained from a Chinese chive field at the Yang Town farm in Shunyi (40°1’ N, 116°6’ E), Beijing, China. Individuals were reared on Chinese chive rhizomes for five generations in an incubator (MLR-352H-PC) at 25 ± 1 °C, 70 ± 5% RH, and 14:10 (L:D).

### 2.2. Effect of Short-Term High-Temperature Stress on Development of Bradysia Odoriphaga Later Stage Larvae and Their Offspring

Sixty third-instar larvae were placed in separate culture dishes (Φ = 60 mm) containing a 2-mm-thick layer of 2.5% solidified agar (CM0131; Oxoid, Basingstoke, UK). The culture dishes were placed in an incubator at one of three temperatures (34, 37, or 40 °C) for one of three exposure times (1, 2, or 4 h), and incubated at 25 °C as a control. The test conditions were set by reference to our previous results [25]. After the exposure, the culture dishes were maintained in an incubator at 25 ± 1 °C, 70 ± 5% RH, and 14:10 (L:D). The larvae were considered to be dead if they did not move when gently touched with a brush at 24-h intervals. Death of larvae was assessed daily. Just after the transformation from larvae into pupae, pupae were counted daily and were moved to 30-mm Petri dishes (containing moistened filter paper). Each pupa was put into a separate Petri dish and was marked. The survival of pupae was monitored daily. Pupae were counted as dead if they did not start eclosion within 10 days at 25 °C. After emergence as adults, unmated male and female adults were still placed in the original 30-mm Petri dish, and their lifespan were recorded daily. Adults were counted as dead if they did not move when gently touched with a brush. The above test was considered as a replicate, and five replicates were used for each combination of temperature and exposure time.

In addition, enough third-instar larvae were treated as above. After emergence as adults, male and female adults (eclosion within 24 h) were paired and placed in individual culture dishes (Φ = 60 mm) as above, with one pair per container. Six pairs were considered as a group. The lifespan of the female and male adults was recorded. The numbers of eggs laid by the female and the hatching rate of those eggs were recorded. If the eggs laid by females in the culture dishes could hatch, it was determined that the pair of male and female adults had mated. The data above were used for statistical analysis. The above test was considered as a replicate, and five replicates were used for each combination of temperature and exposure time.

### 2.3. Data Analysis

SPSS version 17.0 for Windows (SPSS Inc., Chicago, IL, USA) was used for statistical analysis. An inverse sine square-root transformation of the data was performed before analyzing to meet assumptions of normality and homogeneity for parametric analysis. One-way analyses of variance (ANOVA) were used for all comparisons. Treatment effects were considered significant when means were separated with Tukey’s test at *p* < 0.05. Values are expressed as means ± SD.

## 3. Results

### 3.1. Effect of Short-Term High-Temperature Stress on Survival and Pupation of Bradysia Odoriphaga Larvae

**Figure 1:**
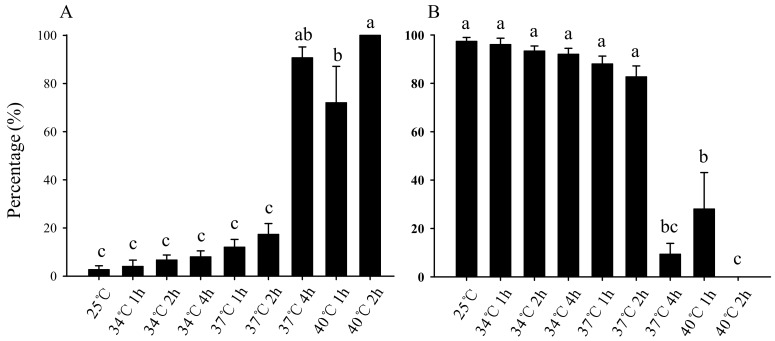
Effect of short-term high-temperature stress on *Bradysia odoriphaga* third-instar larvae first 5 day mortality (**A**) and pupation rate (**B**). The larvae were considered to be dead if they did not move when gently touched with a brush. Just after the transformation from larvae into pupae, pupae were counted daily. Values are means ± SD of five replicates (each of replicate n = 60, total number of each treatment N = 300). Within each panel, bars with different letters are significantly different according to Tukey’s test (*p < 0.05*).

Statistics for larvae after short-term high-temperature stress showed that the average larval mortality in the first 5 days increased with increasing temperature and incubation time. There was no significant difference in the average larval mortality within 5 days after stress treatment at 25 °C or 34 °C for 1 h, 34 °C for 2 h, 34 °C for 4 h, 37 °C for 1 h, and 37 °C for 2 h, with mortality of 2.67%, 4.00%, 6.67%, 8.00%, 12.00%, and 17.33%, respectively. However, after exposure to 40 °C for 2 h, all of the larvae were dead within 5 days, which was not significantly different from the average larval mortality (for 90.67%) within 5 days after stress at 37 °C for 4 h, but significantly higher than the average larval mortality (for 72.00%) after stress at 40 °C for 1 h (Figure 1A).

All of the still-living larvae had pupated, and the average pupation rate decreased with increases in stress temperature and duration. There was no significant difference in the average pupation rate after stress treatment at 25 °C or 34 °C for 1 h, 34 °C for 2 h, 34 °C for 4 h, 37 °C for 1 h, and 37 °C for 2 h, which were 97.33%, 96.00%, 93.33%, 92.00%, 88.00%, and 82.67%, respectively. However, all the larvae died after exposure to 40 °C for 2 h, and the pupation rate was zero, which was not significantly different from the average pupation rate (for 9.33%) of the larvae after exposure to 37 °C for 4 h, but significantly higher than the average pupation rate (for 28.00%) of the larvae after exposure to 40 °C for 1 h (Figure 1B).

### 3.2. Effect of Short-Term High-Temperature Stress on the Bradysia Odoriphaga Pupal Stage

**Figure 2:**
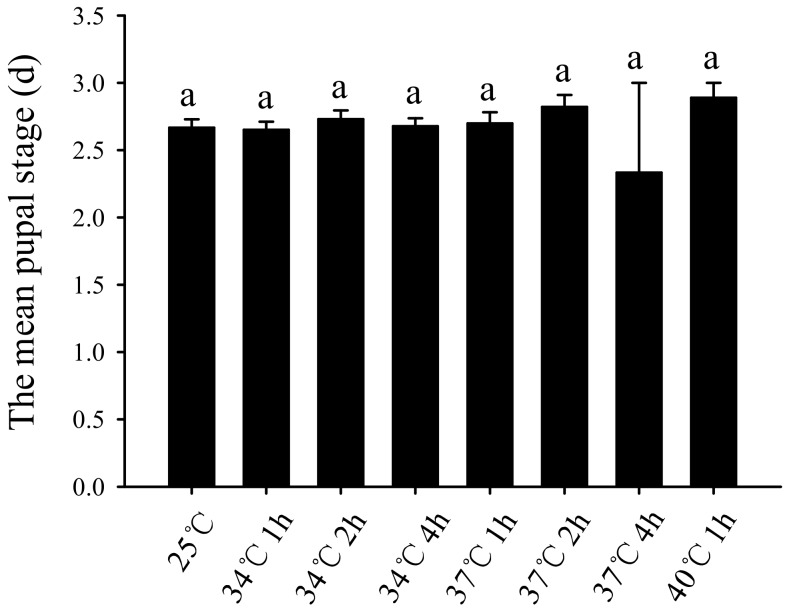
Effect of short-term high-temperature stress on the average pupal stage of *Bradysia odoriphaga* third-instar larvae after pupation ((d) = days). Values are means ± SD of five replicates. Within each panel, bars with same letters are not significantly different according to Tukey’s test (*p ˃* 0.05). Each pupa of per replicate was put into a separate Petri dish and was marked. The change of pupae was monitored daily. The pupal period is the time between pupation and eclosion. Pupae without the capability of eclosion are not included. The total numbers (N) of pupae with the capable of eclosion were 264, 252, 253, 248, 213, 156, 13, and 36 after third-instar larvae exposure to 25 °C or 34 °C for 1 h, 34 °C for 2 h, 34 °C for 4 h, 37 °C for 1 h, 37 °C for 2 h, 37 °C for 4 h, and 40 °C for 1 h, respectively.

After short-term high-temperature treatment, the difference in the subsequent average pupal period was not significant, as long as the *B. odoriphaga* larvae could pupate successfully (Figure 2).

### 3.3. Effect of Short-Term High-Temperature Stress on the Eclosion Rate of Bradysia Odoriphaga Pupae

**Figure 3:**
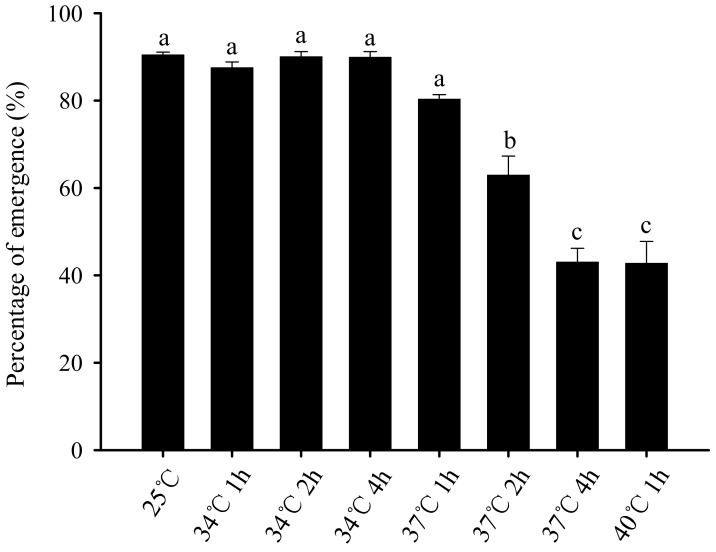
Effect of short-term high-temperature stress on the eclosion rate of subsequent pupae of third-instar *Bradysia odoriphaga* larvae. Values are means ± SD of five replicates. Within each panel, bars with different letters are significantly different according to Tukey’s test (*p < 0.05*). Each pupa of per replicate was put into a separate Petri dish and was marked. The eclosion was monitored daily. Pupae were counted as dead if they did not start eclosion within 10 days. The total numbers (N) of pupae were 292, 288, 281, 276, 265, 248, 29, and 84 after third-instar larvae exposure to 25 °C or 34 °C for 1 h, 34 °C for 2 h, 34 °C for 4 h, 37 °C for 1 h, 37 °C for 2 h, 37 °C for 4 h, and 40 °C for 1 h, respectively.

The average eclosion rate of *B. odoriphaga* pupae decreased with increasing stress temperature and duration (Figure 3). After the larvae were stressed at 25 °C or 34 °C for 1 h, 34 °C for 2 h, 34 °C for 4 h, 37 °C for 1 h, 37 °C for 2 h, 37 °C for 4 h, and 40 °C for 1 h, the eclosion rates of the subsequent pupae were 90.41%, 87.49%, 90.00%, 89.89%, 80.28%, 62.89%, 43.00%, and 42.73%, respectively.

### 3.4. Effect of Short-Term High-Temperature Stress on the Lifespan of Bradysia Odoriphaga Adults

**Figure 4:**
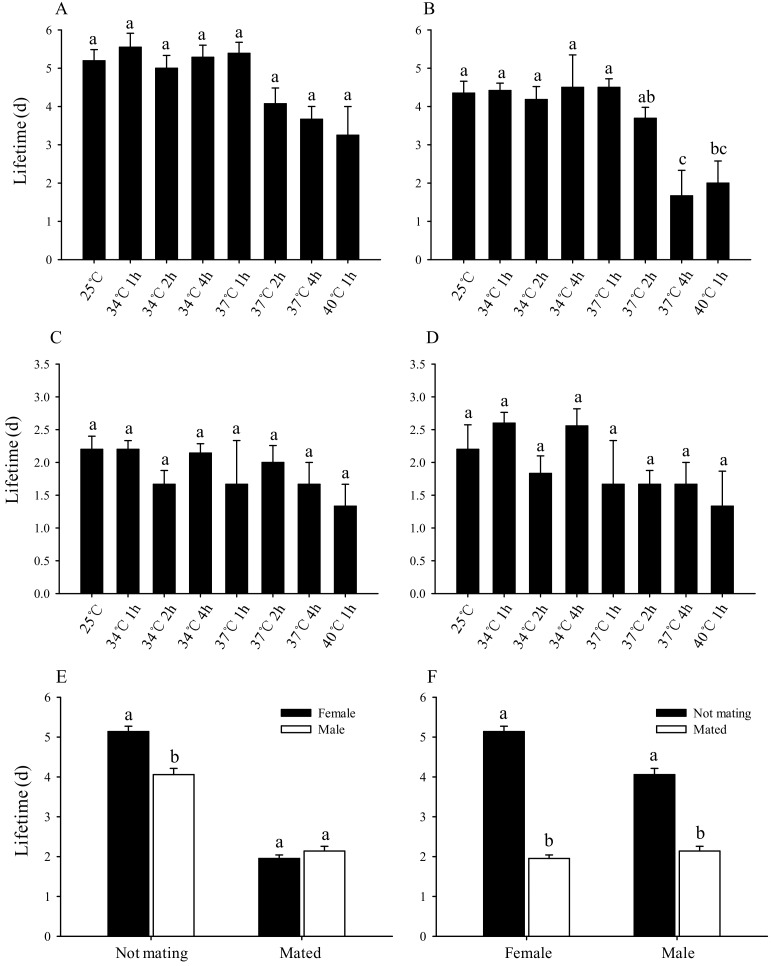
Effect of short-term high-temperature stress on the lifetime of Bradysia odoriphaga adults. (**A**) Unmated female adults, (**B**) unmated male adults, (**C**) mated female adults, (**D**) mated male adults, (**E**) lifespan comparison of male and female adults, and (**F**) lifespan comparison of unmated and mated adults. Values are means ± SD of five replicates. Within each panel, bars with different letters are significantly different according to Tukey’s test (*p* < 0.05) ((d) = days). After emergence as adults, the lifespan of male and female adults was recorded daily. Adults were counted as dead if they did not move when gently touched with a brush. The total numbers (N) of female adults unmated were 143, 128, 133, 124, 105, 76, 7, and 20; the total numbers (N) of male adults unmated were 121, 124, 120, 124, 108, 80, 6, and 16 after third-instar larvae exposure to 25 °C or 34 °C for 1 h, 34 °C for 2 h, 34 °C for 4 h, 37 °C for 1 h, 37 °C for 2 h, 37 °C for 4 h, and 40 °C for 1 h, respectively. The total numbers (N) of female and male adults mated were 30 and 30, respectively, in the different test conditions.

There was no significant difference in the lifespan of the subsequent unmated female adults after third-instar larvae were stressed with short-term high temperatures (Figure 4A). However, the lifespan of the unmated male adults was significantly shortened by increases in stress temperature and duration. For example, there was no significant difference in the subsequent male adult lifespan after larvae exposure to 25 °C or 34 °C for 1 h, 34 °C for 2 h, 34 °C for 4 h, 37 °C for 1 h, or 37 °C for 2 h, which was 4.35 d, 4.42 d, 4.18 d, 4.50 d, 4.50 d, and 3.69 d, respectively. However, the subsequent male adult lifespan was significantly reduced, to 1.67 d and 2 d, when the larvae were stressed at 37 °C for 4 h and 40 °C for 1 h, respectively (Figure 4B).

There was no significant difference in the average lifespan of mating male and female adults after short-term high-temperature stress (Figure 4C,D). The average lifespan of females (5.14 d) was significantly higher than that of males (4.06 d) in unmated adults. The average lifespan of females (1.95 d) was slightly shorter than that of males (2.14 d) for adults after mating, but the difference was not statistically significant (Figure 4E). The average lifespan was significantly shortened for adults after mating, whether female or male (Figure 4F).

### 3.5. Effects of Short-Term High-Temperature Stress on the Oviposition Quantity and Hatching Rate of Subsequent Female Adults

**Figure 5:**
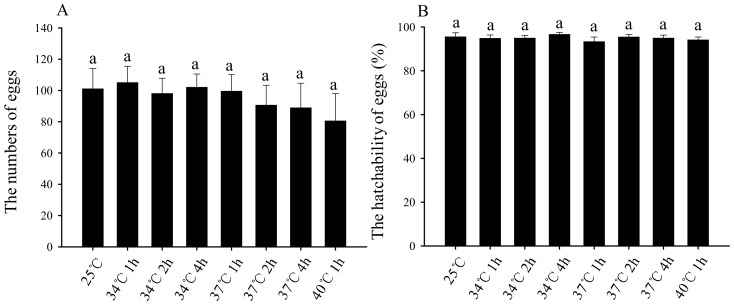
Effects of short-term high-temperature stress of third-instar larvae on oviposition amount (**A**) and hatchability (**B**) in subsequent *Bradysia odoriphaga* female adults. Male and female adults unmated were paired and placed in individual culture dishes (Φ = 60 mm), with one pair per container, and six pairs as a replicate. The numbers of eggs and larvae were recorded. If some of the eggs in a culture dish had hatchability, the numbers of eggs in the culture dish were used for statistical analysis. Values are means ± SD of five replicates. Within each panel, bars with same letters are not significantly different according to Tukey’s test (*p* *˃* 0.05).

After third-instar larvae were treated with short-term high-temperature stress, as long as the larvae can develop into adults normally, the adults can mate and oviposit normally, as differences in average oviposition amount (Figure 5A) and egg hatching rate were not significant (Figure 5B).

## 4. Discussion

Insects are ectotherms. The environmental temperature will affect their survival, distribution, abundance, and life history [27]. Each specific insect has its own optimum survival temperature. Li et al. [8] showed that the optimal developmental temperatures for *B. odoriphaga* range are from 20 °C to 25 °C, with survival declining as temperature increases. Previous research showed that high temperatures can kill insects [28]. When *Leptinotarsa decemlineata* larva was exposed to 65 °C for 10 min, the mortality was 100% [29]. The mortality was 100% when *B. odoriphaga* adults, eggs, larvae, or pupae were exposed to a constant temperature of 40 °C for 1.3, 1.8, 2.8, or 3.7 h, respectively [25]. Even if high temperature does not completely kill particular pests, it can reduce their survival. For example, the mortality rate of *Metopolophium dirhodum* larva was 90% after 33 °C for 8 h [30]. Cheng et al. [26] showed that the fecundity of *B. odoriphaga* generally decreased as temperature and exposure time increased, and no eggs were laid when females were exposed to 37 °C for 2 h. Our study found that the larval mortality was as high as 90.66% after 5 days, although the larvae could not be killed instantly by treatment at 37 °C for 4 h (Figure 1A). These examples provide a theoretical basis for controlling *B. odoriphaga* by using suitably high temperatures. However, when *B. odoriphaga* larvae were treated at 37 °C for 2 h, the mortality was only 17.33% after 5 days. Although this mortality was higher than that of the 25 °C control group (2.67%), the difference was not significant (Figure 1A). It implies that sufficient time at an appropriately high temperature is required for pests to die. Shi et al. [25] showed that the mortality was 100% when *B. odoriphaga* adults, eggs, larvae, and pupae were exposed to constant temperatures of 36 °C for 24.0, 24.0, 48.0, and 48.0 h, respectively. However, it is difficult to maintain 36 °C for 24 h or 48 h in the natural environment, though it is possible to maintain 37 °C for 4 h [31]. Therefore, 37 °C is the critical temperature for controlling *B. odoriphaga* with high temperature. In addition, 100% of the larvae of *B. odoriphaga* died within 5 days of exposure to 40 °C for 2 h, which was not significantly different from the average mortality (90.66%) of larvae exposure to 37 °C for 4 h, but significantly higher than the average mortality (72.00%) of larvae exposure to 40° C for 1 h (Figure 1A). This demonstrates that larval mortality is not only related to high temperature, but also to the duration of high temperature stress. The higher the temperature, the shorter the time it takes to cause larval death, and vice versa [32]. Shi et al. [25] research showed that the mortality was only zero and 64.67% when *B. odoriphaga* larvae were exposed to constant temperatures of 38 °C for 4 h and 40 °C for 2 h, and then were maintained in an incubator at 25 °C for 24 h. However, our result showed that 90.66% and 100% of *B. odoriphaga* larvae died within 5 days of treatment at 37 °C for 4 h and 40 °C for 2 h, respectively. This phenomenon poses a scientific question as to how a series of physiological and biochemical responses had taken place in the larvae of *B. odoriphaga* within 5 days, including some oxidases and heat shock proteins, etc. We will further reveal the heat-stress mechanisms from the physiology, biochemistry, and molecular biology levels in future studies.

This study also found that there was no significant difference in the pupation rate among *B. odoriphaga* larvae that survived 5 d after different short-term high-temperature stress treatment. For instance, the survival rates of the larvae treated at 37 °C for 4 h and 40 °C for 1 h were 9.34% and 28.00%, respectively, and all of the surviving larvae could pupate. In comparison, the survival rate of the control at 25 °C was 97.33%, and all of the surviving larvae could also pupate (Figure 1B). We suggest that 5 days is the critical assessment time. Larvae survival 5 d after the short-term high-temperature treatment indicates that the high temperature treatment cannot be used to control *B. odoriphaga* and will have no impact on subsequent pupation. In addition, there was no significant difference between the pupal periods of different treatment groups (Figure 2). Nevertheless, the eclosion rate of pupae was influenced. Treatments of 25 °C or 34 °C for 1 h, 34 °C for 2 h, 34 °C for 4 h, 37 °C for 1 h, and 37 °C for 2 h had no significant effect on the pupae eclosion rate, while the eclosion rate (for 43.00%) decreased significantly when the temperature was raised to 37 °C and lasted for 4 h (Figure 3). This further indicates that 37 °C for 4 h might be the critical high temperature and duration for *B. odoriphaga* control. Furthermore, this stress condition could also cause secondary damage to the surviving larvae by reducing the eclosion of later stage pupae. This also explains the very low abundance of *B. odoriphaga* in summer in China [6], since days with 37 °C for at least 4 h occur frequently.

Our study also found that there was no significant difference in the lifespan of unmated female adults that emerged from stressed larvae (Figure 4A). However, as the temperature and duration of the short-term stress increased, the lifespan of unmated male adults decreased. For instance, after treatment at 37 °C for 4 h and 40 °C for 1 h, the lifespan of male adults decreased by 2.68 d and 2.35 d, respectively, compared with that at 25 °C (Figure 4B). This suggests that the effect of short-term high-temperature stress on males is greater than that on females. Liang et al. [33] showed that males can mate multiple times during their lifetime. If high temperature stress can significantly shorten the lifespan of males, then this reduces mating. Previous studies have also shown that *B. odoriphaga* relies on female adults to attract male adults to mate and reproduce offspring [34]. If short-term high-temperature stress reduces male lifespans, it will greatly reduce the male opportunities to find females, and thus reduce the population of *B. odoriphaga* offspring. The lifespan of insects is significantly shortened after mating and oviposition [35], consistent with our results. For instance, the average lifespan (1.95 d and 2.14 d) of mated male and female adults of *B. odoriphaga* was shortened by 3.19 d and 1.92 d, respectively, compared with unmated adults (Figure 4F). As a consequence, a control method to consider is releasing large numbers of infertile “virgin” male adults to mate with endemic female adults, so as to shorten the lifespan of fertile female adults and reduce their chances of fruitful reproduction.

The longevity of male and female adults depends on the insect species [36] and its reproductive characteristics [37]. Since living requires energy consumption, insects can extend their lifespan by obtaining energy from nature [38]. However, *B. odoriphaga* adults do not eat and cannot obtain energy. In order to reproduce and leave enough energy for offspring, *B. odoriphaga* look for mates immediately after emerging as imagoes [34]. This study found that the lifespan of unmated female adults was significantly longer than that of unmated male adults (Figure 4E). This may relate to the poor flight ability of females [39], which requires passive acceptance of flying males for mating. Therefore, the extended lifetime of females allows males more opportunities to find them. In addition, the females have a larger body and store much more energy than males [39]—another reason females can live longer than males. Females die soon after they mate and oviposit successfully, as the lifespan of mated females is significantly shorter than that of unmated females. Females only mate once during their lifetime [33], suggesting that their immediate death after oviposition could reduce energy consumption and conserve energy for future generations. In contrast, males can mate up to 13 times during their lifetime [33], which means their reproductive mission has not been completed after mating once, and it is beneficial to continue to search for mates. Therefore, it would be expected that males are less likely to die after mating. However, this study indicates that the life expectancy of mated male adults is only slightly higher than that of female adults, and not statistically significant. It can be inferred that the death of the male may be related to the energy exhaustion following multiple matings. Perhaps, there is no selective advantage for differing lifespans for male and female adults after mating because multiple matings are completed in a short time.

High temperature affects not only the development and reproduction of the affected insects, but also the growth and development of their progeny. For example, high temperatures can affect the growth and development of *Harmonia axyridis* (Pallas) larvae and the fertility of their offspring [40]. The hatchability of *Plutella xylostella* offspring decreased by 20% when adults experienced natural high temperature for 1 d [41]. This phenomenon was also observed in *Grapholitha molesta* [36]. However, our study found that there was no significant effect on the oviposition amount and egg hatching rate for adults resulting from high-temperature-stressed larvae (Figure 5A,B), which suggests that high-temperature stress only affected the contemporary growth and development of *B. odoriphaga* and had no effect on offspring.

Since *B. odoriphaga* larvae mainly live below ground at depth of 0 to 5 cm [6], our results indicate that *B. odoriphaga* could be managed by some methods to keep the soil (0–5 cm in depth) above 37 °C for 4 h, such as soil solarization, plastic films, etc. Soil solarization has been used in the hot season to treat the soil, before planting, in order to eliminate soil-borne diseases and weeds [42,43]. Our previous study firstly reported that when the 0.12-mm-thick light blue anti-dropping film was covered on the soil surface, the soil temperature dependably increased and rapidly killed all stages of *B. odoriphaga* [31]. This study not only provides complementary theoretical support for the technology on soil solarization, but also provides new ideas for the control of other pests. Widespread application of high-temperatures strategies to physically control agricultural pests in the field could produce significant ecological and economic impact [44].

## 5. Conclusions

*B. odoriphaga* is a major biological disaster in Chinese chive industry. This study indicate that the average larval mortality increases with the raise of temperature and prolongation of exposure time. Non-lethal high temperature at 37 °C for 4 h can hinder development and allow control of *B. odoriphaga*. Therefore, artificially raising the soil temperature over 37 °C for 4 h in the living spaces of *B. odoriphaga*, would be a promising strategy for environmentally-friendly pest control.
